# Personalization of ^99m^Tc-sestamibi activity in SPECT/CT myocardial perfusion imaging with the cardiofocal SmartZoom® collimator

**DOI:** 10.1186/s40658-023-00545-8

**Published:** 2023-03-24

**Authors:** Emilie Verrecchia-Ramos, Olivier Morel, Valérie Beauchat, Sylvie Denet, Abdourahamane Djibo Sidikou, Merwan Ginet, Estelle Pfletschinger, Luminita Teodor, Maud Trombowsky, Jeany Verdier, Christelle Vère, Paul Retif, Sinan Ben Mahmoud

**Affiliations:** 1 CHR Metz-Thionville, Department of Medical Physics, Mercy Hospital, 1, Allée du Château, 57530 Ars-Laquenexy, France; 2 CHR Metz-Thionville, Department of Nuclear Medicine, Mercy Hospital, 1, Allée du Château, 57530 Ars-Laquenexy, France; 3 CHR Metz-Thionville, Department of Nuclear Medicine, Bel-Air Hospital, 1, Rue du Friscaty, 57100 Thionville, France; 4 Université de Lorraine, CNRS, CRAN, 54000 Nancy, France

**Keywords:** Myocardial perfusion imaging, Single photon emission computed tomography, Nuclear medicine, Anger, Radiation protection

## Abstract

**Background:**

Patient radioprotection in myocardial perfusion imaging (MPI)-SPECT is important but difficult to optimize. The aim of this study was to adjust injected activity according to patient size—weight or BMI—by using a cardiofocal collimator camera.

**Methods:**

The correlation equation between size and observed counts in image was determined in patients who underwent stress Tc-99m-sestamibi MPI-SPECT/CT with a cardiofocal collimator-equipped conventional Anger SPECT/CT system. Image quality analyses by seven nuclear physicians were conducted to determine the minimum patient size-independent observed count threshold that yielded sufficient image quality for perfusion-defect diagnosis. These data generated an equation that can be used to calculate personalized activity for patients according to their size.

**Results:**

Analysis of consecutive patients (*n* = 294) showed that weight correlated with observed counts better than body mass index. The correlation equation was used to generate the equation that expressed the relationship between observed counts, patient weight, and injected activity. Image quality analysis with 50 images yielded an observed count threshold of 22,000 counts. Using this threshold means that the injected activity in patients with < 100 kg would be reduced (e.g., by 67% in 45-kg patients). Patients who are heavier than 100 kg would also benefit from the use of the threshold because although the injected activity would be higher (up to 78% for 150-kg patients), good image quality would be obtained.

**Conclusions:**

This study provided a method for determining the optimal injected activity according to patient weight without compromising the image quality of conventional Anger SPECT/CT systems equipped with a cardiofocal collimator. Personalized injected activities for each patient weight ranging from 45 to 150 kg were generated, to standardize the resulting image quality independently of patient attenuation. This approach improves patient/staff radioprotection because it reduces the injected activity for < 100-kg patients (the majority of patients).

**Supplementary Information:**

The online version contains supplementary material available at 10.1186/s40658-023-00545-8.

## Background

Myocardial perfusion imaging (MPI) in single-photon emission computed tomography (SPECT) is a widely used technique for assessing coronary artery disease [1]. However, it can constitute a significant source of population ionizing radiation. Thus, an important consideration in MPI-SPECT is patient and staff radioprotection.

MPI-SPECT has long been performed on Anger SPECT systems equipped with low-energy high-resolution (LEHR) parallel hole collimators [2, 3]. Due to the limited detection sensitivity of this system [4], acquisition of the stress image during the 1-day stress–rest protocol necessitates the administration of approximately 400 MBq [5–7]. According to the Summary of Product Characteristics published by the French agency ANSM [8], this stress activity yields an effective dose of 3.2 mSv. A few hours later, the rest image is acquired after administering threefold more activity: this high level is needed to cover the residual signal from the preceding stress test. This rest activity yields an effective dose of 10.8 mSv [8]. Consequently, the total effective dose for conventional SPECT systems equipped with LEHR collimators reaches 14 mSv [9].

However, several years ago, SPECT system technological developments produced semiconductor detector modules [2] that directly detect the gamma rays. The small size of these cadmium zinc telluride (CZT) detectors facilitated the development of heart-dedicated camera designs that optimize detection efficiency. This has dramatically reduced the injected activity: for example, a 50-kg patient undergoing exercise CZT SPECT now only requires 64 MBq (0.48 mSv) of the ^99m^Tc-sestamibi radiotracer [10].

**Fig. 1:**
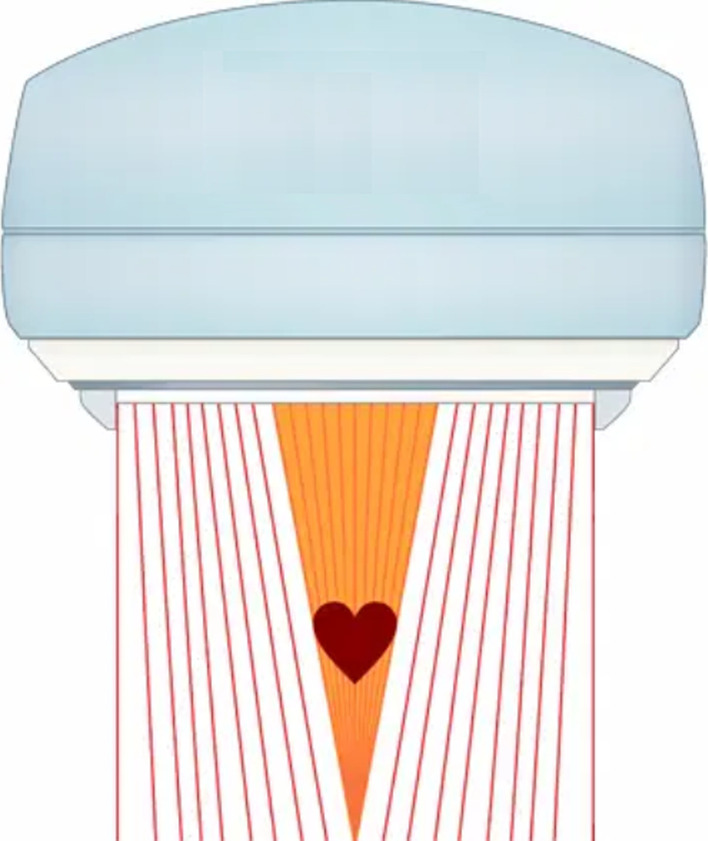
Schematic depiction of the geometry of the cardiofocal SmartZoom collimator used in this study

The main drawback of the cardiac CZT cameras is they are not suitable for purposes other than measuring cardiological activity, which makes their substantial cost prohibitive. Indeed, most nuclear medicine departments are preferentially equipped with multipurpose cameras. In this case, an interesting solution is to use a cardiofocal collimator, which increases detection sensitivity without limiting the applications of the camera [2, 4, 11]. This kind of collimator was defined by Hawman and Haines in 1994 [11] and uses a geometry that is intermediate to those employed by the parallel hole and fan collimators. Specifically, the angle of the septa varies continuously such that they converge in the central region at the heart but increasingly adopt a parallel orientation toward the edge of the collimator. Figure 1 is based on an image in the commercial brochure of Siemens that depicts the SmartZoom® collimator [4].

Two studies have shown that when a cardiofocal collimator is used instead of a LEHR collimator, the injected activity is reduced by up to 67% [12] or a factor of four [13]. However, neither study proposed how to tailor the injected activity to the individual patient, nor did they determine the degree of patient size-related gamma-ray signal attenuation. Notably, Van Dijk et al. showed that when this kind of optimization was applied to conventional LEHR collimators, it effectively reduced the tracer activity in lean patients without compromising image quality [14–16].

The present study therefore sought to optimize patient and staff radioprotection during MPI-SPECT with a cardiofocal collimator-equipped conventional Anger camera by tailoring the injected ^99m^Tc-sestamibi activity to patient weight or body-mass index (BMI).

## Methods

### Study design

First, the variable “myocardial signal” (MS) was defined as the average count in the myocardial region of interest (ROI) in the MPI-SPECT image. Second, the MS in stress MPI-SPECT images of a large patient cohort was determined retrospectively. The rest images were not analyzed because the threefold additional activity that is injected at rest leads to high signal-to-noise ratios in any case. The analysis of the stress images yielded an equation that described the relationship between MS and patient weight (Eq. 1). Third, Eq. 1 is used to derive an equation that described the relationship between MS, patient weight, and injected activity over a constant scan time (Eq. 2). Fourth, the MS threshold that indicated acceptable image quality for perfusion-defect diagnosis was determined by asking seven nuclear physicians to evaluate the quality of a subset of the cohort stress images. Fifth, to display the utility of our approach, the MS threshold was plugged into Eq. 2 to generate a table listing the optimal activity (or scan time) for patients with specific body weights.

### Patient selection and MPI-SPECT imaging

The cohort consisted of 294 adult patients who underwent an exercise or pharmacological stress test and ^99m^Tc-sestamibi MPI-SPECT with a cardiofocal collimator-equipped camera. It was a consecutive series that included healthy patients and patients with perfusion defects. It was used to derive the equation between body weight and MS; a subset (*n* = 50) was also used to evaluate image quality.

In accordance with the clinical procedures in our institution, the ^99m^Tc-sestamibi activity in all patients was ~ 260 MBq. Acquisition was performed with a conventional large-field dual-head Anger gamma-camera Intevo Bold (Siemens Healthineers) equipped with the SmartZoom® cardiofocal collimator [4] and the IQ-SPECT cardio-centered acquisition process [17]. The acquisition parameters are specified in Table 1. Scan time was fixed to 20 s per projection angle, using 17 projections. After manual reorientation of the input tomograms to achieve optimal separation of the left and right ventricle activity, the 4.8-mm cubic voxel size SPECT images were reconstructed with an iterative 3-dimensional ordered subsets expectation maximization (OSEM3D) algorithm (10 iterations, 3 subsets), a 10-mm full-width at half-maximum (FWHM) Gaussian filter, and a mask to exclude any surrounding activity (for example, that from gastrointestinal uptake). Only the uncorrected (UC) images were analyzed in this retrospective study. This was to avoid potential bias caused by the attenuation correction [18]. Moreover, it is likely that obtaining good quality UC images will further improve the corrected images. In addition, using the UC images is consistent with the clinical habits in our institution and others, which is to work with both UC and corrected images.

**Table 1:** Clinical acquisition parameters

Total examination duration, min	≈ 15
Orbit of detectors (min–max)	104°, cardio-centric (239°–343°)
Number of frames	17
Frame acquisition duration, sec	20
Matrix size	128 × 128
Zoom	1
Position	Supine, holding their arms above their head to minimize attenuation
Energy window (width, %—center, keV)	15%, 140 keV
ECG-gated, number of gates	Yes, 16 gates
Associated low-activity tomodensitometry for attenuation correction	Yes

ECG, electrocardiogram

### Definition of myocardial signal and its relationship with patient weight or BMI

**Fig. 2:**
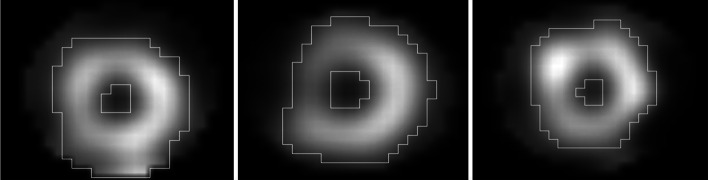
Myocardial ROI in the uncorrected clinical SPECT images of three patients. The ROI was defined as the pixels whose counts exceeded 45% of the maximal pixel value

For each stress test UC MPI-SPECT image, an ROI corresponding to the myocardial area (*i.e.* the MS) was defined as the pixels whose counts exceeded 45% of the maximal pixel count in the whole image. Since a 10-mm FWHM Gaussian filter is applied to the reconstructed image, the maximal pixel is subject to very little noise and is an accurate representation of the actual tracer fixation in the myocardium. The 45% threshold was empirically chosen to obtain the best correlation between the ROI counts and patient weight or BMI. Figure 2 shows examples of these ROIs in three patients.

The MS of the 294-patient cohort was plotted as a function of patient weight or BMI, and the relationship between these variables was determined empirically by power fitting. To assess whether weight or BMI correlated better with MS at a statistical level, the means of the absolute relative error (%) in each model were compared by paired t-test (alpha level 0.05). To confirm that MS is associated significantly with patient weight, one-way analysis of variance (ANOVA) comparing the weight deciles in the total cohort in terms of MS was conducted, after verifying the following assumptions: independent and random observations, normality, and homoscedasticity.

### Identification of a MS threshold for sufficient image quality for diagnosis

The relationship between MS and the quality of the clinical MPI-SPECT images was determined by extracting the UC stress test SPECT images of 50 patients from the 294-patient cohort. These patients were selected so that this subcohort had a high frequency of heavy patients (48% had BMI > 30 kg/m^2^), who had poor quality images due to gamma attenuation (Table 2). The images were subjected to independent visual evaluation by seven senior nuclear physicians without any additional information about the patient. After examining each image, each physician scored the quality of the image. The maximal score was 100, meaning that the image quality was perfect, while the lowest score was 0, which signified an unusable image. The 50 images were then categorized into good (score > 66), intermediate (score 33–66), and poor (score < 33) image-quality subgroups. To determine the MS threshold above which the image quality is sufficient, we studied the distribution of the MS in the poor/intermediate image quality images *versus* the good image quality images by constructing box-and-whisker plots. The first quartile of the good-quality images was taken as the optimal MS threshold because this meant that 75% of the good-quality images had an MS count that exceeded this threshold.

**Table 2:** Demographic and treatment characteristics of the patient cohorts

Variable	Total cohort used to derive the equation *n* = 294^a^	Subgroup used for image-quality evaluation *n* = 50^a^
Age, years	66 ± 10	65 ± 11
Women, *n* (%)	105 (36)	15 (30)
Height, cm	170 ± 9	172 ± 10
Weight, kg	84 ± 17	92 ± 22
Weight range, kg (min–max)	41–145	44–145
BMI, kg/m^2^	29 ± 6	31 ± 7
BMI > 30 (obese), *n* (%)	109 (37)	24 (48)
BMI range (min–max)	17–53	18–53
Injected activity, MBq	259 ± 13	257 ± 12

The data are expressed as mean ± standard deviation unless otherwise indicated

BMI, body mass index

^a^ The 294-patient cohort was used to derive the equation between MS and body weight while the 50-patient subgroup of the latter cohort was used for image quality evaluation

## Results

In total, 294 consecutive patients were included. This cohort thus represents our clinical habits. It was used to derive the equation between body weight and MS, while a selected subgroup (*n* = 50) was used for image-quality analysis. Table 2 summarizes the main characteristics of these cohorts.

### Cohort analysis showing the relationship between MS and patient weight or BMI

**Fig. 3:**
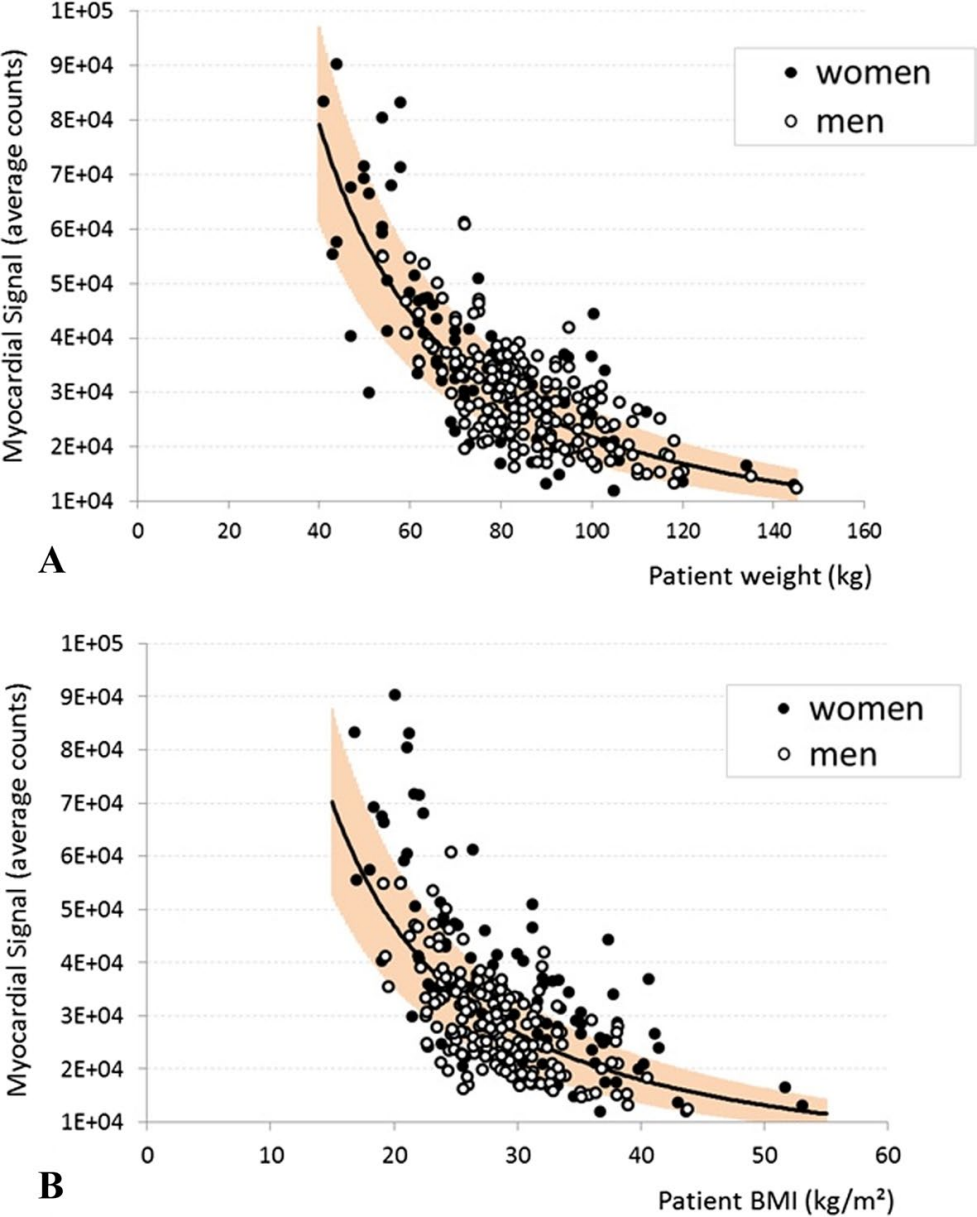
Relationship between myocardial signal and patient weight (**A**) or BMI (**B**). Women and men are shown by black and white circles, respectively. Myocardial signal is the average count in the myocardial ROI. The black line indicates the best fitting curve for the whole cohort. The orange area indicates the confidence intervals of the predicted count numbers that were derived from the fitting curve. BMI, body-mass index; ROI, region of interest

The relationships between MS and patient weight or BMI in the 294-patient cohort are shown in Fig. 3. The best fitting curves were obtained with the equation *y* = *ax*^*b*^. The *a*, *b*, and correlation coefficients are shown in Table 3. In the total cohort and the female and male subgroups, weight correlated better with MS than BMI (*r*^2^ = 0.57 vs. 0.46). Comparison of the average absolute relative errors between the measured and predicted MS values for weight and BMI (20.0 vs. 24.8%) with paired *t* test showed that this difference was statistically significant (*p* < 0.001). Thus, patient weight was chosen for activity adjustment. The equation (Eq. 1) expressing the weight:myocardial signal relationship in this cohort was:

**Table 3:** Correlations between patient weight or BMI and myocardial signal

Dependent variable	Independent variable ^a^	Sex	*a* coefficient	*b* coefficient	Determination coefficient *R*^2^	ANOVA *p* value
Myocardial signal	Patient weight	Women & men	1.4 × 10^7^	− 1.41	0.57	9 × 10^–46^
		Women only	1.7 × 10^7^	− 1.45	0.60	
		Men only	1.2 × 10^7^	− 1.36	0.50	
	Patient BMI	Women & men	3.0 × 10^6^	− 1.39	0.46	
		Women only	4.9 × 10^6^	− 1.49	0.58	
		Men only	2.4 × 10^6^	− 1.34	0.41	

ANOVA, analysis of variance; BMI, body mass index

^a^These correlations were measured in all patients in the 294-patient cohort, or only the women or men

1 $$\mathrm{MS}=14.1\times {10}^{6}\times {\mathrm{weight}}^{-1.41}$$

Analysis of variance (ANOVA) confirmed the significant relationship between MS and body weight (*p* value = 9 × 10^–46^).

### Proposition for tailoring the activity to individual patient weight

The cohort analysis revealed an empirical relationship between the MS in the SPECT image and patient weight (Eq. 1). Separate analyses with a cardiac phantom confirmed that dead-time losses were negligible and that the MS in the SPECT image bore a linear relationship with the equivalent patient activity (data not shown, available in Additional file 1). These observations allowed us to derive Eq. 2, which describes how to maintain a constant MS by tailoring the activity (or scan time, as it is interchangeable with activity [14, 16]) based on a nominal activity of 260 MBq and a nominal scan time of 20 s per projection (17 projections) (these parameters correspond to our clinical habits):

2 $$\mathrm{MS}=14.1\times {10}^{6}\times {\mathrm{weight}}^{-1.41}\times \frac{\mathrm{tailored \, activity}}{\mathrm{nominal\, activity}}=14.1\times {10}^{6}\times {\mathrm{weight}}^{-1.41}\times \frac{\mathrm{tailored\, scan\, time}}{\mathrm{nominal \,scan \,time}}$$

### Identification of a MS threshold that associates with acceptable image quality

**Fig. 4:**
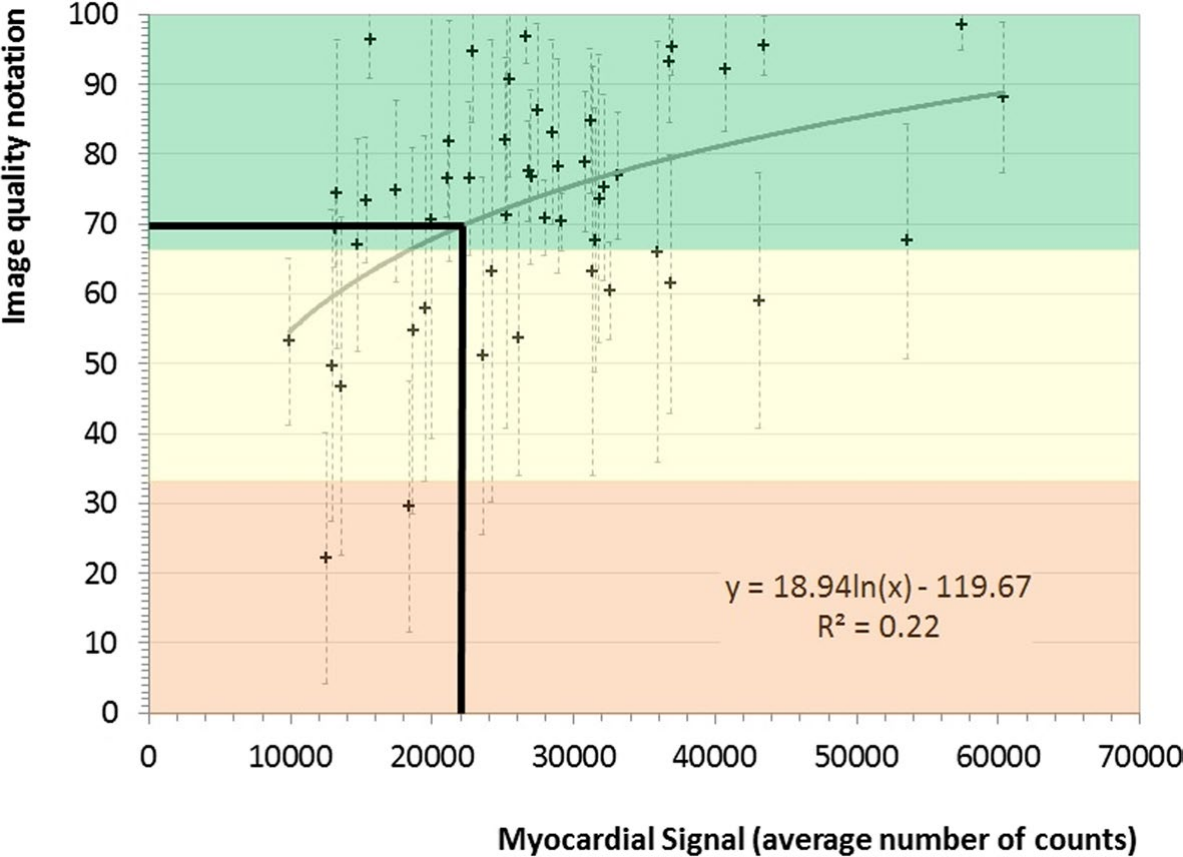
Image quality of the uncorrected stress-test SPECT images of the 50-patient subcohort. The image quality at different myocardial signals was scored by seven nuclear physicians. Black crosses and gray error bars, respectively, indicate the average and standard deviation of the image quality scores recorded by the physicians. The orange, yellow, and green areas indicate poor (score < 33), intermediate (score 33–66), and good (score > 66) image quality, respectively. The gray curving line represents the logarithmic regression of the data. The black landmarks show how the chosen MS threshold of 22,000 counts corresponds to the expected image quality. An image quality score of around > 70 signifies good image quality

**Fig. 5:**
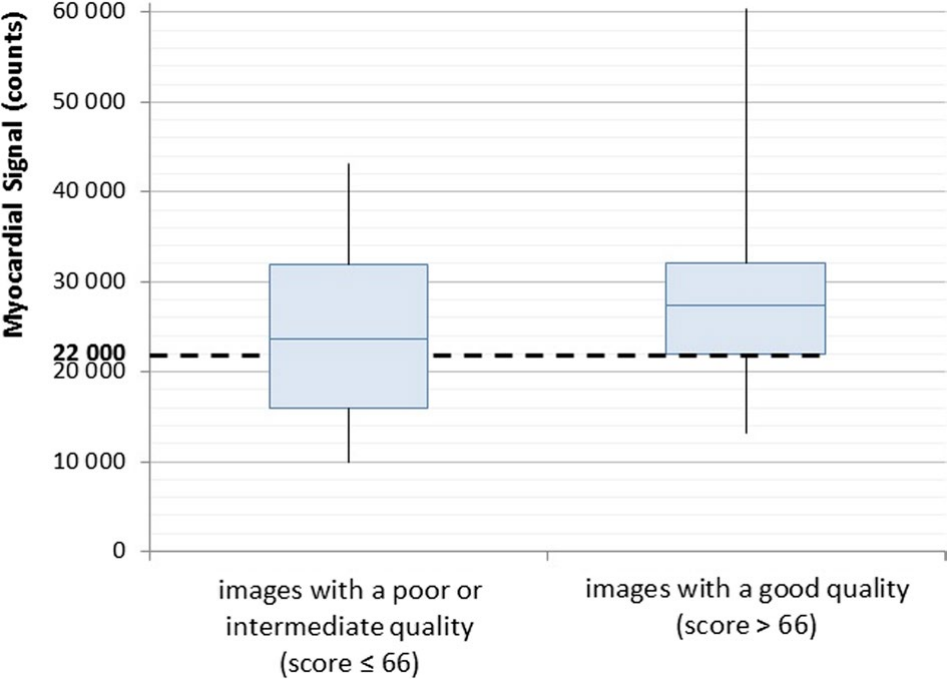
Box plot showing the myocardial-signal distributions in the poor-/intermediate- and good-quality images. The myocardial signal at the first quartile of the good-quality images was 22,000 counts. It was chosen as the myocardial signal threshold

As shown in Fig. 4, the image quality scores given by the nuclear physicians improved as the MS count increased. The images were grouped into good (score > 66)-, intermediate (score 33–66)-, and poor (score < 33)-image quality subgroups and the poor-/intermediate- and good-quality images were compared in terms of MS distribution (Fig. 5). This allowed us to define optimal MS as 22,000 counts (*i.e.,* the first quartile in the good-quality subgroup). Figure 4 shows the quality score as a function of MS: the logarithmic regression of the data confirms that one can expect good quality images in most cases when a 22,000-count MS threshold is applied.

**Fig. 6:**
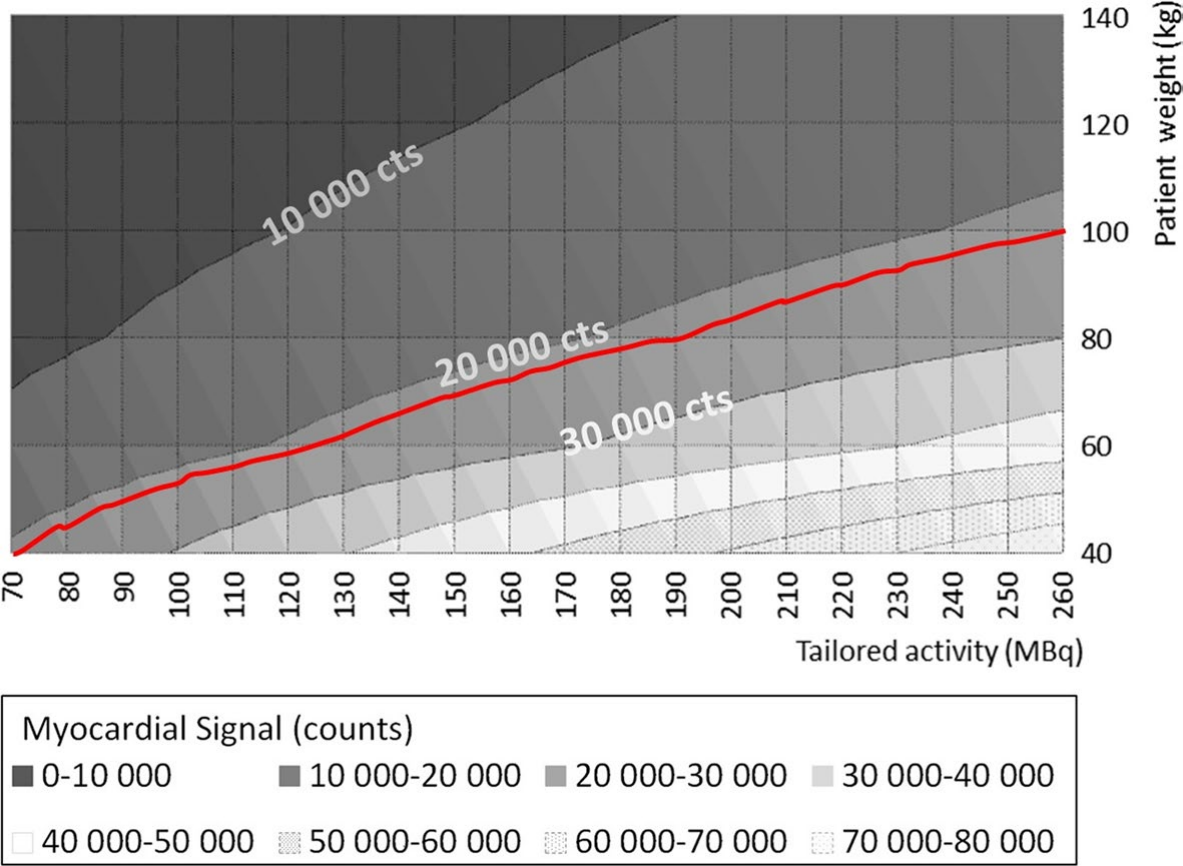
Diagram depicting the dual-dependency relationship between myocardial signal, patient weight, and injected activity. The theoretical expected myocardial signal was computed using Eq. 2. Uncertainty derived from Eq. 2 leads to a constant relative uncertainty of 23% over the predicted myocardial signal. Applying a myocardial signal threshold of 22,000 counts (red line) means that patients with a body weight of < 100 kg will benefit from activity reduction

The MS threshold can be plugged into Eq. 2 together with patient weight to easily determine the activity that should be injected to obtain the target MS of 22,000 counts. Figure 6 shows the relationships between MS, patient weight, and injected activity. Equation 2 can also be used to identify the scan time that should be used to acquire the target MS. Table 4 lists the injected activity and scan times that correspond to the 22,000-count MS threshold for different patient weights.

**Table 4:** Examples of patient weight-based personalization of optimal activity and scan time

Patient weight, kg	Personalized activity using a fixed scan time (20 s per projection angle, using 17 projections), MBq	Personalized scan time using a fixed activity (260 MBq), seconds	Relative difference^a^ in activity or scan time compared to clinical practice (260 MBq or 20 s per projection angle, respectively), %
45	85	7	− 67
70	159	12	− 39
100	260	20	0
120	338	26	+ 30
150	462	36	+ 78

^a^Negative relative differences indicate a reduction in activity or scan time, whereas positive relative differences indicate increased activity or scan time

Figure 6 and Table 4 both indicate that the MS threshold of 22,000 counts can significantly reduce the injected activities in patients with a body weight below 100 kg. The amount of reduction is dependent on body weight: thus, while patients around 100 kg will benefit from activity reduction of just a few percentage points, patients with a weight of 45 kg can be injected with a 67% lower activity than the current standard activity without compromising the image quality. By contrast, for patients over 100 kg, our results show that greater injected activity (or scan time) will be needed to maintain a constant image quality. This increase can reach 78% of nominal conditions for the heaviest patients (150 kg).

## Discussion

This study proposes a method for tailoring the activity during MPI-SPECT with cardiofocal collimator-equipped conventional Anger SPECT/CT systems according to the body weight of individual patients (Eq. 2). When this equation is applied, patients with a low body weight of 45 kg can benefit from a 67% reduction in administered activity, which represents a significant lowering of the exposure to medical ionizing radiation. Moreover, our approach has the advantage that it will also generate good-quality images with heavy patients (> 100 kg): even though these patients will be administered with more activity than our current standard (a 150-kg patient will receive 78% more), this will yield the imaging quality needed for accurate diagnosis. Thus, our approach complies with ALARA recommendations [19] in all patients.

The minimal MS was chosen as the first quartile in the good-quality subgroup images from a survey conducted with seven physicians. This implies that the general activity administered (260 MBq) is already too high in 75% of the patients. This can be explained by the fact that our current practice is to administer 260 MBq to all patients regardless of their weight, and this activity was chosen to achieve images of sufficient quality from most patients (including the heavier patients). Therefore, it was expected that on balance, 75% of patients (those < 100 kg) would receive too much activity.

To confirm our results, it will be necessary to conduct further research exploring the reliability with which the images obtained with the optimized activities lead to the correct diagnosis. This complementary work will be performed in a further prospective study. In any case, our study shows how a personalized body weight activity equation can be derived and how the impact of activity reduction on clinical images can be investigated. Notably, our findings are supported by van Dijk et al. [14–16]: they showed that a body weight-dependent protocol for MPI with conventional SPECT systems effectively reduced the tracer activity in lean patients. As an aside, the MS threshold used in our study (22,000 counts) was determined from the myocardial ROI in the reconstructed images. It cannot be compared to the 1 million count threshold estimated by Nakazato et al. [20] because that threshold was determined from the planar projections for a completely different imaging device (CZT dedicated cardiac camera).

This study has some limitations. First, as mentioned, a validation study is needed to prove the diagnostic reliability of our approach. Second, when conducting the image-quality analysis, we noted a considerable inter-observer variability in terms of image quality scores (Fig. 4). This reflects the subjective nature of this scoring system and the multitude of factors that can affect image quality, such as patient position, the centering and reorientation of the myocardium, and patient metabolism (e.g., digestive uptake), and this enhances the need for a validation study. Third, we chose to optimize the UC images rather than the corrected images. We chose this approach because if the optimized UC images are of sufficiently good quality, the corrected images are likely be of even higher quality, thus promoting accurate diagnoses. Moreover, this approach limits possible bias induced by attenuation correction such as over-correction of the data and introduction of artifacts [18]. In addition, the use of UC images is consistent with the clinical habits of our institution and others, who work with both UC and corrected images. Finally, the reconstruction parameter adaptation to more noisy projections was not investigated. However, this is because the current parameters are already chosen to reduce the noise level: it is unlikely that increasing the FWHM Gaussian filter that is applied to the reconstructed images will reduce the noise further without impairing the spatial resolution.

We should mention here the Artificial Intelligence (AI) algorithms that were recently proposed for de-noising nuclear medicine images [21] and specifically for optimizing the MPI dose [22]. These techniques are based on deep-learning applied to complex neural networks and may be able to significantly reduce the administrated activity without degrading image quality [22]. Although their reliability in terms of de-noising images remains to be validated clinically, we believe these AI approaches are very promising. In particular, they may be able to substantially complement the dose-to-weight adjustment approach that we propose: while our method takes into account the amount of attenuation induced by each patient, the AI methods can lower the noise level in all images. Thus, both techniques together could significantly improve MPI-SPECT outcomes.

## Conclusions

This activity optimization study confirmed that patient weight can be used to tailor the injected activity for conventional Anger cameras equipped with a cardiofocal collimator. We observed that a 22,000-count MS threshold significantly reduces the activity in all patients under 100 kg, thereby improving patient and staff radioprotection. In heavier patients, the activity will have to be increased, but nonetheless the patient will benefit from good-quality imaging. Thus, our approach complies with ALARA recommendations. We are currently conducting a prospective study to confirm that this approach does not affect the diagnostic reliability of MPI-SPECT. Indeed, given that heavier patients will have better images, it is possible that our approach could even improve overall diagnostic accuracy.

## Supplementary Information

**Additional file 1.** Detector linearity and MS reproducibility measured with a heart and thorax phantom.

## Abbreviations

AC: Attenuation correction
AI: Artificial Intelligence
BMI: Body mass index
CZT: Cadmium zinc telluride
ECG: Electrocardiogram
LEHR: Low energy high resolution
MPI: Myocardial perfusion imaging
MS: Myocardial signal, defined as the average number of counts in the myocardial ROI
ROI: Region of interest
SPECT: Single-photon emission computed tomography
UC: Uncorrected

## Declarations

### Availability of data and materials

The datasets used and/or analyzed during the current study are available from the corresponding author on reasonable request.
