# Supplementary material for: Personalization of 99mTc-sestamibi activity in SPECT/CT myocardial perfusion imaging with the cardiofocal SmartZoom® collimator
Source: EJNMMI Phys. 2023 Mar 24;10:23. doi: 10.1186/s40658-023-00545-8 (PMC10036680; doi:10.1186/s40658-023-00545-8)
Supplement: Supplementary file 1 — Additional file 1. Detector linearity and MS reproducibility measured with a heart and thorax phantom. [file 40658_2023_545_MOESM1_ESM.docx]

# [Original RESEARCH] [EJNMMI PHYSICS]

# Personalization of 99mTc-Sestamibi activity in SPECT/CT myocardial perfusion imaging with the cardiofocal SmartZoom® collimator: Supplementary Data

Emilie Verrecchia-Ramos, PhD ^a*^, Olivier Morel, MD ^b^, Valérie Beauchat, MD ^c^, Sylvie Denet, MD ^b^, Abdourahamane Djibo-Sidikou, MSc ^a^, Merwan Ginet, MD ^b^, Estelle Pfletschinger, MSc ^a^, Luminita Teodor, MD ^b^, Maud Trombowsky, BSc ^a^ ,Jeany Verdier, PharmD ^b^, Christelle Vère, MD ^c^, Paul Retif, PhD ^a,d^ and Sinan Ben Mahmoud, MD, MSc ^b^

^a^ CHR Metz-Thionville, Department of Medical Physics, Mercy Hospital, 1, allée du château, 57530 Ars-Laquenexy, FRANCE

^b^ CHR Metz-Thionville, Department of Nuclear Medicine, Mercy Hospital, 1, allée du château, 57530 Ars-Laquenexy, FRANCE

^c^ CHR Metz-Thionville, Department of Nuclear Medicine, Bel-Air Hospital, 1, rue du Friscaty, 57100 Thionville, FRANCE

^d^ Université de Lorraine, CNRS, CRAN, F-54000 Nancy, FRANCE

* Correspondence: e.verrecchiaramos@chr-metz-thionville.fr

*This supplementary document describes the measurements performed on a heart phantom in order to check the negligibility of dead time effect and the linear response of the detector with the activity in the field of view.*

**Phantom Measurements: Methods**

A thorax phantom was assembled from a 20 cm-diameter cylindrical water phantom. To mimic myocardial uptake, an 8 cm-diameter spherical compartment in the thorax phantom was filled with a ^99m^Tc solution and maintained at the center of the water cylinder.

To study the relationship between MS and equivalent patient activity, we conducted 21 acquisitions using the clinical IQ-SPECT workflow, with various activities of ^99m^Tc ranging between 3.9 and 1.95 MBq in the spherical myocardial-mimicking compartment, which represent the myocardial uptake in humans after 260–130 MBq is injected, respectively.

After acquisition of the image-set, the myocardial ROI was defined as described above for the patient images and we analyzed the effect on MS of decreasing ^99m^Tc activities.

**Phantom Measurements : Results**

Figure 1 shows that MS had a linear relationship with activity (r²=0.99). The excellent correlation coefficient indicates that there is no dead-time effect to consider at this activity range, and MS is highly reproducible with the myocardial ROI definition of 45% of maximal pixel count.


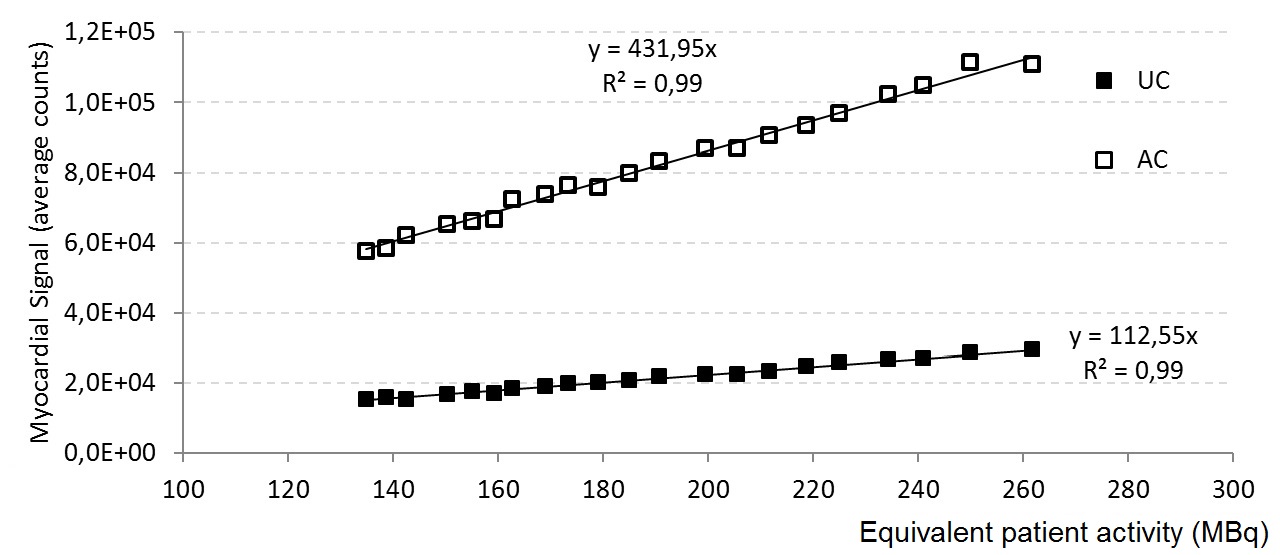


**Fig. 1 Myocardial signal in the myocardial compartment of the phantom for different equivalent patient activities.** Myocardial signal was defined as the average number of counts in the myocardial compartment ROI. UC: uncorrected images; AC: attenuation-corrected images
